# Sexual orientation disparities in food insecurity and food assistance use in U.S. adult women: National Health and Nutrition Examination Survey, 2005–2014

**DOI:** 10.1186/s12889-020-09261-9

**Published:** 2020-08-13

**Authors:** Joanne G. Patterson, Jennifer Russomanno, Jennifer M. Jabson Tree

**Affiliations:** 1 The Ohio State University Comprehensive Cancer Center, c/o College of Public Health, 1841 Neil Avenue, 400A Cunz Hall, Columbus, OH 43110 USA; 2 University of Tennessee Graduate School of Medicine, 1924 Alcoa Highway, Box U94, Knoxville, TN 37920 USA; 3 University of Tennessee Department of Public Health, 367 HPER, Knoxville, TN 37996 USA

**Keywords:** Sexual and gender minorities, Sexual minority women, Lesbian, Bisexual, Food insecurity, Food assistance, Health status disparity, Minority health

## Abstract

**Background:**

Nearly 40 million American adults report past year food insecurity. This is concerning, as food insecurity is associated with chronic disease morbidity and premature mortality. Women disproportionately experience food insecurity, and sexual minority women (i.e., lesbian, bisexual, and heterosexual women reporting same-sex behavior; SMW) may be at greater risk for experiencing food insecurity disparities. The purpose of this study was to investigate patterns and prevalence of food insecurity and food assistance use in sexual minority and exclusively heterosexual women using population-level health surveillance data.

**Methods:**

Using pooled 2004–2014 National Health and Nutrition Examination Survey data (*N* = 7379), we estimated weighted point prevalence of past 12-month food insecurity, severe food insecurity, Supplemental Nutrition Assistance Program (SNAP) use, and emergency food assistance use. We then used Poisson regression with robust variance to estimate prevalence ratios comparing SMW to exclusively heterosexual women on all outcomes. Women were classified by sexual identity and lifetime same-sex behavior as lesbian (*n* = 88), bisexual (*n* = 251), heterosexual and reporting same-sex behavior (heterosexual WSW; *n* = 366), or exclusively heterosexual women (referent; *n* = 6674).

**Results:**

Between 20.6–27.3% of lesbian, bisexual, and heterosexual WSW reported past 12-month food insecurity (versus 13.1% of exclusively heterosexual women). All SMW reported greater prevalence of past 12-month food insecurity and severe food insecurity than exclusively heterosexual women: prevalence ratios (PR) ranged from 1.34 (95% confidence interval [CI], 1.05–1.70) to 1.84 (95% CI, 1.13–3.01). No differences were found in SNAP participation by sexual orientation, but more lesbians and heterosexual WSW reported using emergency food assistance in the past 12-months (PR = 1.89; 95% CI, 1.29–2.79 and PR = 1.43; 95% CI, 1.03–2.00 respectively).

**Conclusions:**

All SMW reported higher prevalence of food insecurity than exclusively heterosexual women. Lesbians and heterosexual WSW were also more likely to rely on emergency food assistance. This is problematic as SNAP use may reduce food insecurity over time, but emergency food resources (e.g., food pantries) do not. More evidence is needed to understand the multilevel factors driving food insecurity in this population to develop policy and community-based efforts to increase SNAP participation and decrease food insecurity.

## Background

Food security, defined as “access by all people at all times to enough food for an active, healthy life” [1], is a leading determinant of poor health. In 2018, approximately 37.2 million Americans—or 11.1% of the population—were food insecure [1]. Of these, almost 39% (9.5 million people) reported very low food security—meaning that household members experienced disrupted eating patterns and reduced food intake (heretofore, severe food insecurity) [1]. Women are on average 10% more likely to experience food insecurity than men [2]. This gender disparity is concerning, as food insecurity is associated with multiple leading causes of death and disability— including cancer, chronic obstructive pulmonary disease, stroke, and diabetes [3, 4]—and associated risk factors (e.g., poor nutrition, obesity, smoking, and chronic inflammation [4, 5]). Given this evidence, addressing food insecurity in women is a public health priority.

### Food insecurity in sexual minority women

An estimated 2.2–6.7% of adult women (7–22 million) in the United States (US) identify as sexual minority (e.g., lesbian, gay, or bisexual) [6]. Given published food insecurity rates—where, in 2018, 11.1% of the general US population reported experiencing food insecurity—we estimate that 777,000 to 2.4 million sexual minority women (SMW) experience food insecurity annually. This is especially concerning as food insecurity is associated with greater annualized health care expenditures [7]. Using National Health Interview Survey data, Berkowitz and colleagues estimated that food insecure individuals had an additional $1863 in health care expenditures annually [7]. If this is true for SMW, food insecurity could result in $1.45–4.47 billion in excess health care costs for this population each year. Alarmingly, these numbers likely underestimate food insecurity prevalence in SMW.

Warnecke’s multilevel model of population health suggests that discriminatory social conditions lead to inequitable distribution of resources and subsequent health disparities [8]. For SMW, heterosexist and homophobic social conditions are theorized to deplete social and economic resources—including employment, wages, and social connections—resulting in inequitable distribution of health risks, including food insecurity. As such, we hypothesize that SMW are more likely to experience food insecurity than exclusively heterosexual women (i.e., women who identify as heterosexual and report exclusively heterosexual sexual behavior). However, empirical findings about food insecurity in SMW are inconsistent. Gallup survey data indicates that compared to heterosexual women, more lesbian, gay, bisexual, and transgender (LGBT) women report not having enough money to buy food over the past year (20% vs. 34%, *p* < .05) [9]. However, in a population-based study using National Health Interview Survey data, SMW were not more likely than heterosexual women to experience food insecurity during the past 30-days [10]. These mixed findings may reflect differences in how food insecurity is measured. Previous studies have not used the United States Development of Agriculture (USDA) recommended, multidimensional measure of food insecurity [11], which includes a 12-month time assessment to document multiple facets of food insecurity. There is evidence that food security fluctuates across seasons [12] and is pronounced in vulnerable, low-income groups due to employment variability [13] and cost variations (e.g., heating/cooling costs) [14]. Consequently, the period during which a survey is distributed across the year may differentially capture food insecurity in respondents. To better ascertain the breadth of SMW’s experiences with food insecurity, studies using comprehensive measures of food security with longer recall periods are needed.

### Use of food assistance

It is relatively unknown how SMW interact with and utilize the two primary modes of food assistance in the US—the federal Supplemental Nutrition Assistance Program (formerly referred to as “food stamps”; SNAP) and community-based emergency food assistance (e.g., food pantries, soup kitchens). SNAP is a means-tested program that provides food assistance to participating low- and no- income households [15]. In 2017, approximately 42.2 million people—13% of the population—received SNAP benefits [16]. Previous studies using population-level data indicate that sexual minority adults are 1.33–1.73 times more likely than heterosexual adults to receive federal food assistance [9, 10]. There is also evidence that SNAP participation is not equally distributed across SMW. Using National Survey of Family Growth data, both Gates [9] and Brown [10] determined that bisexual women participated in SNAP at higher rates than heterosexual women (28–34% versus 18–24%), but no differences were observed for lesbians (19–32%).

Other strategies for alleviating food insecurity include use of community-based emergency food assistance—including food pantries and soup kitchens. These resources are not means-tested and are generally provided by nonprofit religious or civic groups [17]. As such, reliance on emergency food assistance is commonplace among vulnerable populations—including women [18]. However, given historical and contemporary discrimination experienced by sexual minorities from religious organizations [19, 20], SMW may be less likely to access emergency food assistance. To our knowledge, no published studies assess disparities in emergency food assistance participation in SMW.

This study investigated prevalence and disparities in past 12-month food insecurity and use of food assistance by subgroups of SMW. We hypothesized, per Warnecke’s model [8], that more SMW would experience food insecurity and severe food insecurity than exclusively heterosexual women. In accordance with previous publications, we hypothesized that SMW would be more likely than exclusively heterosexual women to use SNAP, but that SMW would be less likely than exclusively heterosexual women to report using emergency food assistance.

## Methods

This study was a secondary analysis of de-identified data and did not require a human subject’s review.

### Study design

Publicly available data from the National Health and Nutrition Examination Survey (NHANES) were pooled across 10 years, 2005–2014, for this study. NHANES is a national probability, repeated cross-sectional survey of US adults and children > 12 years old that assesses health and nutrition status using interviews and medical examinations [21]. Gender is assessed by interviewers during the household screening such that respondents are assigned “male” or “female” gender based on either physical characteristics or direct inquiry. Transgender-inclusive gender identity was neither asked nor recorded. Respondents characterized as “female” during the interview comprise the sample of women in our study.

**Fig. 1:**
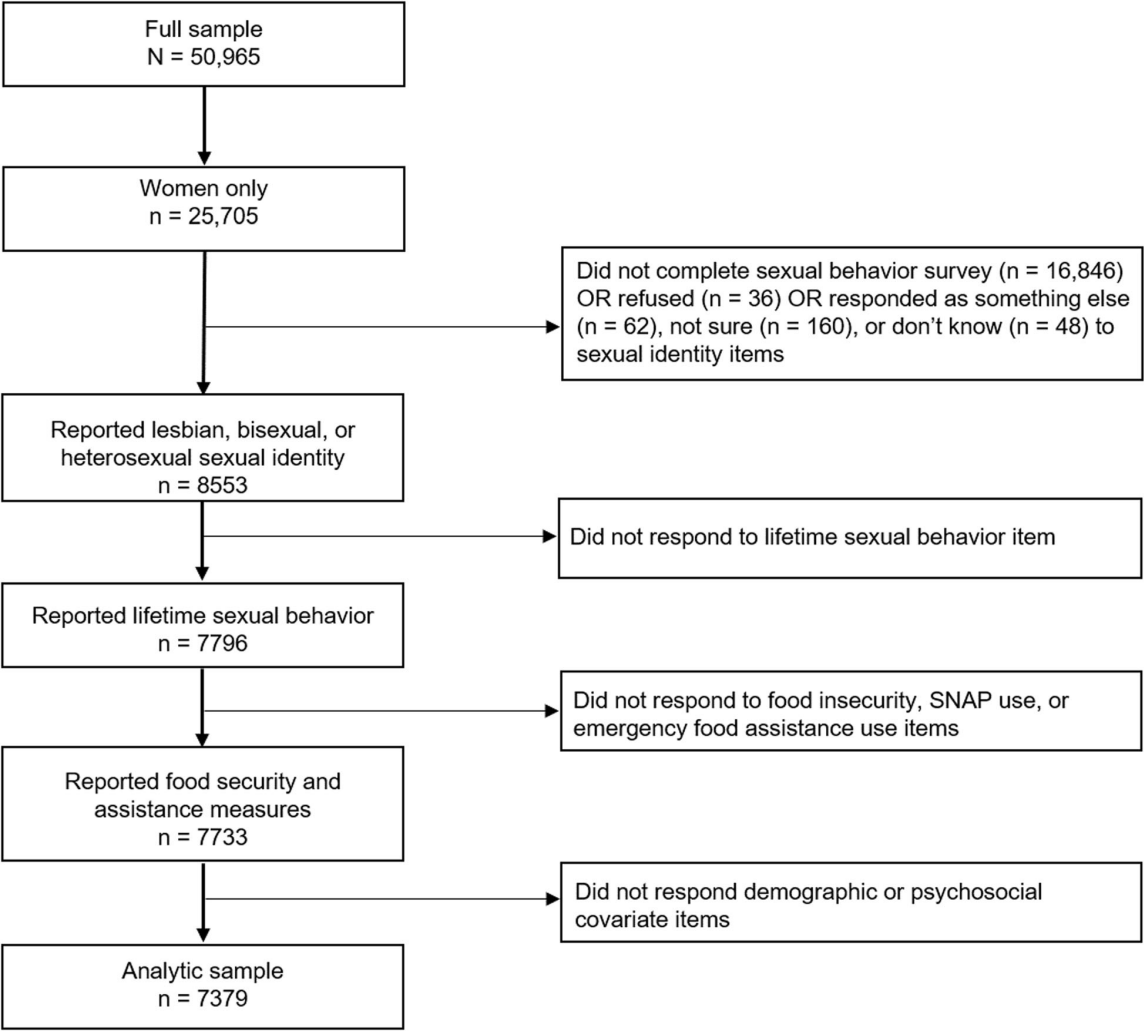
Flowchart of exclusions for deriving the analytic sample

NHANES data vary across survey years such that some data (e.g., alcohol use) are not publicly available for the subsample of respondents < 20 years old at time of interview. Moreover, some sexual orientation questions (e.g., sexual identity) are not asked of women > 60 years old at time of interview. Consequently, our study sample included women aged 20–59 who completed sexual behavior surveys. Respondents were excluded from analyses if they did not answer sexual identity, lifetime same-sex sexual behavior, food security, alcohol use, and tobacco use questions. The final analytic sample included 7379 women (Fig. 1).

### Dependent variables

#### Food insecurity

NHANES uses the USDA’s US Household Food Security Survey Module (α = 0.74–0.93 [22]) to assess past 12-month food insecurity. This measure assesses food insecurity across 4 domains, including: (1) anxiety about food supplies, (2) perceptions that quality or quantity of food is not adequate, and reduced food intake by (3) adults or (4) children (if applicable). Food security is assessed using a scale of 0–10 for households without children and 0–18 for households with children. Levels of household (HH) food security are designed as “full food security” (0 points), “marginal food security” (1–2 points), “low food security” (3–5 points HH without child, 3–7 points HH with child), and “very low food security” (6–10 points HH without child, 8–18 points HH with child). Variables were recoded so that individuals were considered food insecure if scores were ≥ 3 (low or very low food security; coded 1) and food secure if scores were ≤ 2 (i.e., full or marginal food security; coded 0) [23]. For sensitivity analyses, the variables were recoded so that individuals were considered severely food insecure if scores were ≥ 6 (household without child) or ≥ 8 (household with child) (very low food security; coded 1) and food secure (i.e., full marginal, or low food security; coded 0) if scores were ≤ 5 (household without child) or ≤ 7 (household with child).

#### Receipt of SNAP benefits

Respondents affirming that they, or another household member, were authorized to receive or received food stamp/SNAP benefits in the past 12-months were coded as receiving SNAP (coded 1) versus those not authorized to receive/did not receive past 12-month food stamp/SNAP benefits (coded 0).

#### Receipt of emergency food assistance

Emergency food assistance was assessed with the question, “In the last 12 months, did [you/you or any member of your household] ever get emergency food from a church, a food pantry, or a food bank, or eat in a soup kitchen?” Respondents were coded as receiving emergency food assistance in the past 12 months (coded 1) or not receiving past 12-month emergency food assistance (coded 0).

### Independent variables

Sexual orientation was defined in terms of sexual identity and sexual behavior according to best practice [24] and previous publications [25]. Women aged 18–59 years were asked, “Do you think of yourself as heterosexual or straight (i.e., sexually attracted only to men); homosexual or gay (i.e., sexually attracted only to women); bisexual (i.e., sexually attracted to men and women); something else?”. Women were also asked to report the number of women and men with whom they had engaged in sexual behavior over the life course. We defined women’s sexual orientation as follows: Women reporting heterosexual identity and lifetime sexual activity with only male partners were defined as exclusively heterosexual women (coded 0). Women identifying as lesbian and reporting any lifetime sexual activity with women were defined as lesbian women who have sex with women (lesbian WSW; coded 1). Women identifying as bisexual and reporting any lifetime sexual activity with women were defined as bisexual WSW (coded 2). Women identifying as heterosexual and reporting any lifetime sexual activity with women were defined as heterosexual WSW (coded 3).

### Covariates

Summary statistics were calculated to describe demographic, socioeconomic, and psychosocial factors. Age was recoded into four categories representing respondents across emerging (18–25), young (26–35), middle (36–45), and mid-late (46–59) stages of adulthood. NHANES’ original variable structure was retained for race/ethnicity categories (non-Hispanic white, non-Hispanic black, Mexican American, other Hispanic, and other race including multiracial). Education level was recoded into three categories (< high school/General Education Diploma (GED), some college/Associate’s degree, college graduate or higher). In multivariable analyses, race/ethnicity was dichotomized into person of color (coded 1) and not a person of color (coded 0). Education was dichotomized into < high school/GED (coded 1) and > high school/equivalent degree (coded 0). Family poverty to income ratio was calculated by dividing family income by the Health and Human Services Poverty guidelines specific to family size, year and state [22]. For descriptive analyses, family poverty to income ratio was presented by US Census defined poverty thresholds (< 100%, 100–199%, 200–299%, 300–399, > 400%). For regression analyses, family poverty to income ratio was dichotomized where respondents were considered poor (income < 200% federal poverty level [FPL]; coded 1) or not poor (income ≥200% FPL; coded 0). For summary statistics, health insurance was defined as reporting private insurance, Medicare/Medigap, Medicaid, other public insurance, or being uninsured. In multivariable analyses, we defined health insurance coverage as private (coded 0), public (coded 1), or none/uninsured (coded 2). Alcohol use [26] and cigarette smoking [27, 28] are two psychosocial characteristics that are associated with food insecurity and are known disparities in SMW [29]. Women were defined as at-risk drinkers (coded 1) if, during the past 12 months, they reported having > 7 or more drinks per week [30]. Current cigarette smoking was defined as having smoked > 100 cigarettes ever and currently reporting smoking on either “some” or “every” day (coded 1).

### Analyses

Summary statistics, including weighted proportions and unweighted cases, described the sample. We assessed differences in the distribution of sociodemographic and psychosocial variables between sexual minority and heterosexual respondents using Likelihood Ratio chi-squared test for proportions (LR X2). We then used weighted bivariate analyses with LR X2 test for proportions to investigate differences in the point prevalence of food insecurity and food assistance use across sexual orientation subgroups. Results were reported as weighted percentages with associated 95% confidence intervals, test statistics, and *p*-values. To calculate the relative risk of food insecurity, severe food insecurity, SNAP participation, and emergency food assistance use in SMW (versus exclusively heterosexual women), we estimated prevalence ratios and associated 95% confidence intervals using Poisson regression with robust variance estimation. This method has been used in previous studies with LGBT populations where the outcomes of interest are common (i.e., > 10%) [31, 32] such that other analytic methods (e.g., logistic regression) may overestimate the prevalence ratio [33–35]. Covariates selected a priori as potential confounders included age, race/ethnicity, income, educational attainment, health insurance coverage, risky drinking, and smoking. Multivariable analyses were adjusted for survey year to account for potential unmeasured cohort effects. Sampling weights based on the NHANES multistage design were used for all multivariable models. We used the “subpop” command for variance estimation with Taylor series linearization as per NHANES guidance [21]. STATA 16.0 (StataCorp LP, College Station, TX) was used for all analyses.

## Results

Table 1 summarizes sample demographic, socioeconomic, and psychosocial characteristics. Of respondents, 1.2% were lesbian WSW (*n* = 88), 3.4% bisexual WSW (*n* = 251), and 5.1% heterosexual WSW (*n* = 366).

**Table 1:** Sample characteristics in women, by self-reported sexual orientation: National Health and Nutrition Examination Survey, 2005–2014

	Exclusively heterosexual women	Lesbian WSW	Bisexual WSW	Heterosexual WSW		
	Weighted % (unweighted n)				X2	*P*
Total	90.4 (6674)	1.2 (88)	3.4 (251)	5.1 (366)		
Race/Ethnicity					3.7	<.001
White, non-Hispanic	67.3 (2891)	69.1 (42)	70.3 (127)	73.7 (201)		
Black, non-Hispanic	12.1 (1419)	19.3 (28)	16.7 (72)	12.5 (84)		
Hispanic	14.2 (1800)	7.6 (12)	8.5 (35)	8.1 (55)		
Multiple races	6.4 (564)	4.1 (6)	4.5 (17)	5.7 (26)		
Age					10.9	<.001
20–25	13.3 (989)	14.0 (20)	36.4 (86)	19.1 (71)		
26–35	23.0 (1671)	18.1 (18)	28.9 (84)	24.1 (101)		
36–45	26.4 (1788)	41.5 (26)	16.7 (44)	28.3 (95)		
46–59	37.3 (2226)	26.4 (24)	18.0 (37)	28.5 (99)		
Educational Level					3.1	0.008
< High school	33.2 (2652)	24.4 (28)	42.6 (112)	26.9 (112)		
Some college/AA degree	34.9 (2288)	33.7 (34)	36.1 (97)	43.8 (97)		
College graduate or above	31.9 (1734)	41.9 (26)	21.3 (42)	29.3 (92)		
% Federal Poverty Level					3.4	<.001
< 100%	41.6 (2136)	37.2 (26)	25.2 (50)	32.9 (97)		
100–199%	14.1 (801)	13.6 (10)	10.1 (21)	15.1 (42)		
200–299%	13.3 (835)	8.1 (8)	13.8 (33)	14.1 (53)		
300–399%	16.8 (1493)	23.8 (22)	26.2 (72)	22.6 (92)		
> 400%	14.2 (1409)	17.3 (22)	24.7 (75)	15.3 (82)		
Insurance Type					5.3	<.001
Private	66.7 (3730)	52.8 (35)	49.0 (93)	55.2 (173)		
Medicare/Medigap	1.3 (109)	6.5 (6)	0.21 (1)	2.5 (10)		
Medicaid	6.8 (690)	2.6 (4)	11.9 (45)	11.1 (66)		
Other public	6.7 (525)	6.4 (5)	6.9 (21)	7.1 (23)		
None	18.5 (1620)	31.7 (38)	32.0 (91)	24.2 (94)		
Risky Drinker	45.7 (2902)	64.5 (57)	66.8 (169)	60.2 (219)	22.0	<.001
Current Smoker	20.4 (1341)	41.6 (38)	46.3 (129)	37.3 (13)	37.6	<.001

*X2* Likelihood ratio chi-squared; *P* = *p*-value

There were substantial differences in demographic, socioeconomic, and psychosocial characteristics between heterosexual and sexual minority women (Table 1). Most SMW in this sample identified as non-Hispanic White or non-Hispanic Black. Moreover, bisexual WSW were significantly younger than exclusively heterosexual women. Bisexual WSW were less likely to have graduated college than their exclusively heterosexual counterparts. All SMW were more likely to report at-risk drinking and current smoking. Lesbian and bisexual WSW were more likely to report having no health insurance.

**Fig. 2:**
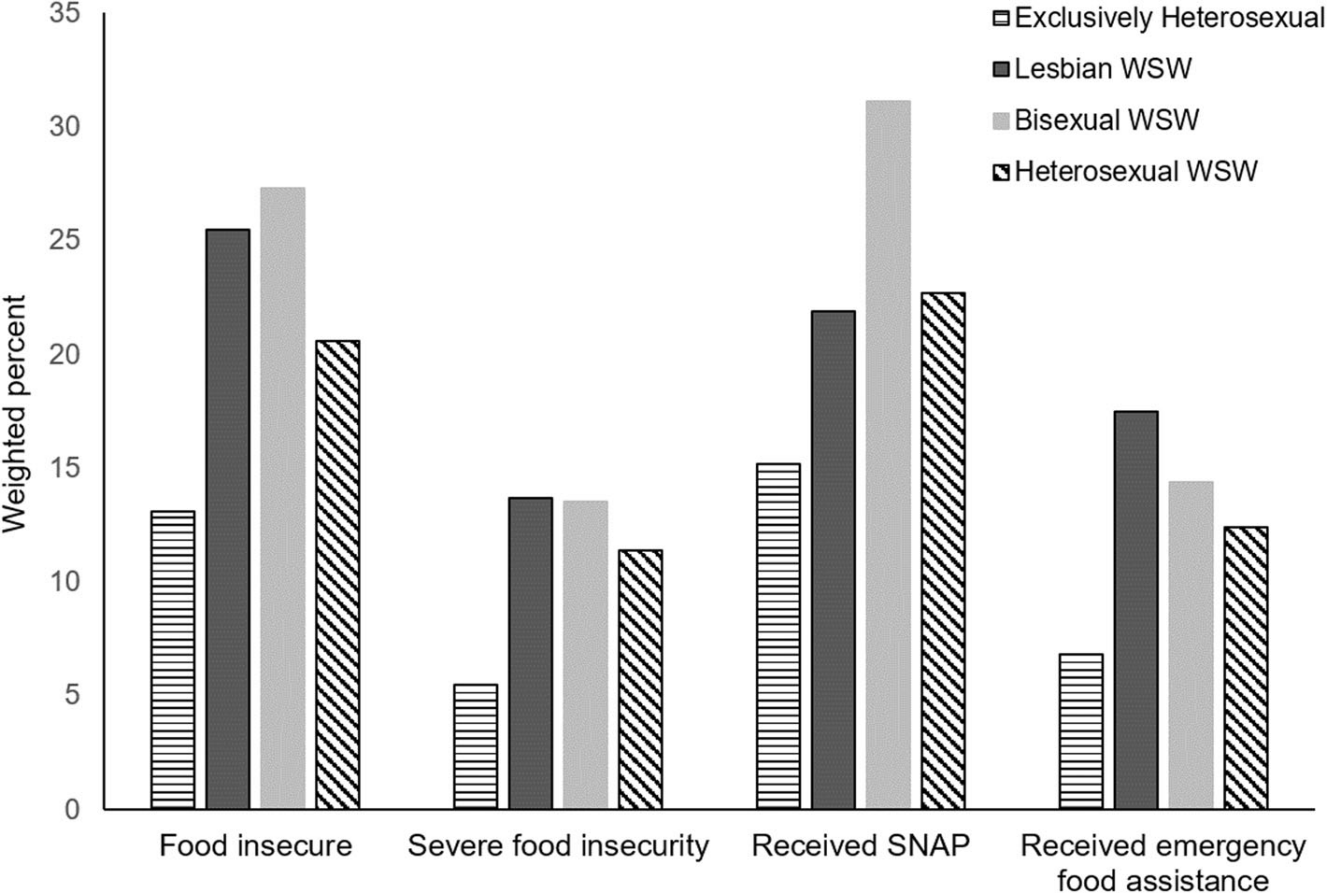
Prevalence of past 12-month food insecurity and food assistance use in women, by sexual orientation

Table 2 reports weighted point prevalence estimates of food insecurity and food assistance use. Over 1 in 5 SMW reported experiencing food insecurity in the past 12-months, with bisexual WSW reporting the highest estimated prevalence (27.3%; 95% CI, 21.09–34.61) followed by lesbian WSW (25.5%; 95% CI, 16.60–36.97). More than 1 in 7 lesbian and bisexual WSW were reported severe food insecurity during the past 12-month (13.7%; 95% CI, 7.94–22.7 and 13.5%; 95% CI, 9.40–19.02, respectively). Over 1 in 4 SMW reported using SNAP during the past 12-months; however, estimated prevalence of SNAP use was highest for bisexual WSW (31%; 95% CI, 24.9–38.06). Lesbian WSW reported highest prevalence of using emergency food assistance (17.5%; 95% CI, 10.31–28.09) (Fig. 2).

**Table 2:** Weighted prevalence of food insecurity and food assistance use among women in the US, by self-reported sexual orientation: NHANES 2005–2014 (*n* = 7379)

	Exclusively Heterosexual	Lesbian WSW	Bisexual WSW	Heterosexual WSW		
	Weighted % (95% CI)				X2	*P*
Food insecure	13.1 (11.94–14.27)	25.5 (16.60–36.97)	27.3 (21.09–34.61)	20.6 (15.98–26.02)	13.8	<.001
Severe food insecurity	5.5 (4.75–6.26)	13.7 (7.94–22.7)	13.5 (9.40–19.02)	11.4 (5.42–6.92)	13.4	<.001
Past 12-month SNAP use	15.2 (13.59–17.02)	21.9 (12.9–34.59)	31.1 (24.9–38.06)	22.7 (18.44–27.54)	13.9	<.001
Past 12-month emergency food assistance	6.8 (5.85, 8.00)	17.5 (10.31, 28.09)	14.4 (10.38–19.67)	12.4 (6.50, 18.65)	11.0	<.001

*95% CI* 95% Confidence Interval, *X2* Likelihood ratio chi-squared; *P* = *p*-value

Table 3 reports fully adjusted models estimating prevalence of food insecurity and food assistance use. Both lesbian WSW and bisexual WSW were more likely to report experiencing food insecurity than exclusively heterosexual women (lesbian WSW: PR = 1.52; 95% CI, 1.05–2.20 and bisexual WSW: PR = 1.34; 95% CI, 1.05–1.70). Disparities were also evidenced for heterosexual WSW, who were 35% more likely to experience food insecurity than exclusively heterosexual women (PR = 1.35; 95% CI, 1.05–1.70). The magnitude of the effect was greater in sensitivity analyses. All SMW were more likely to report experiencing severe food insecurity over the past 12-months (lesbian WSW: PR = 1.84; 95% CI, 1.13–3.01; bisexual WSW: PR = 1.50; 95% CI, 1.04–2.16; heterosexual WSW: PR = 1.68; 95% CI, 1.20–2.36).

**Table 3:** Fully-adjusted prevalence ratios for food insecurity and food assistance use for sexual minority compared to exclusively heterosexual women in the US: NHANES 2005–2014 (*n* = 7379)

	Food Insecurity	Severe food insecurity	SNAP use	Emergency food assistance use
	PR (95% CI)			
Sexual Orientation				
Lesbian WSW	1.52* (1.05–2.20)	1.84* (1.13–3.01)	1.13 (0.74–1.73)	1.89** (1.29–2.79)
Bisexual WSW	1.34* (1.05–1.70)	1.50* (1.04–2.16)	1.12 (0.94–1.24)	1.36 (0.97–1.89)
Heterosexual WSW	1.35* (1.05–1.72)	1.68** (1.20–2.36)	1.20 (0.98–1.47)	1.43* (1.03–2.00)
Exclusively heterosexual	Ref	Ref	Ref	Ref
Age				
20–25	1.06 (0.90–1.26)	0.91 (0.68–1.20)	1.21* (1.03–1.41)	0.65*** (0.51–0.83)
26–35	1.02 (0.88–1.19)	1.01 (0.79–1.30)	1.32*** (1.17–1.48)	0.85 (0.71–1.03)
36–45	1.16 (0.97–1.39)	1.16 (0.87–1.56)	1.03 (0.88–1.19)	0.95 (0.78–1.17)
46–59	Ref	Ref	Ref	Ref
Race/Ethnicity				
Person of color	1.40*** (1.20–1.62)	1.36** (1.10–1.69)	1.23*** (1.09–1.38)	1.06 (0.85–1.31)
Non-Hispanic White	Ref	Ref	Ref	Ref
Education level				
< High school	1.18** (1.05–1.32)	1.05 (0.86–1.26)	1.38*** (1.25–1.51)	1.03 (0.87–1.22)
> High school	Ref	Ref	Ref	Ref
Income				
< 200% FPL	3.60*** (3.06–4.24)	4.75*** (3.54–6.39)	4.14*** (3.42–5.02)	4.24*** (2.99–6.02)
> 200% FPL	Ref	Ref	Ref	Ref
Health Insurance				
Public	1.87*** (1.62–2.16)	1.66*** (1.34–2.07)	3.80*** (3.10–4.65)	3.43*** (2.54–4.62)
None	1.67*** (1.43–1.96)	1.43** (1.10–1.84)	2.30*** (1.92–2.77)	2.67*** (1.93–3.68)
Private	Ref	Ref	Ref	Ref
Risky alcohol user				
Yes	0.93 (0.83–1.03)	1.66 (1.34–2.07)	3.80*** (3.10–4.65)	3.43** (2.54–4.62)
No	Ref	Ref	Ref	Ref
Current smoker				
Yes	1.46*** (1.27–1.69)	1.89*** (1.50–2.39)	1.47*** (1.31–1.65)	1.78*** (1.45–2.18)
No	Ref	Ref	Ref	Ref
Survey Year	1.10*** (1.06–1.16)	1.14*** (1.07–1.22)	1.12*** (1.08–1.18)	1.05 (0.97–1.13)

*PR* Prevalence ratio, *95% CI* 95% Confidence Interval. Exclusively heterosexual women served as the referent group to lesbian WSW, bisexual WSW, and heterosexual WSW. *P < .05 ** *P* < .01 ***P < .001

No differences were evidenced between SMW and heterosexual women in prevalence of receiving past 12-month SNAP benefits. Differences were indicated in receipt of emergency food assistance. Lesbian WSW were 89% more likely to report using emergency food assistance than exclusively heterosexual women (PR = 1.89; 95% CI, 1.29–2.79) and heterosexual WSW were 47% more likely (PR = 1.43; 95% CI, 1.03–2.00).

## Discussion

### Food insecurity in SMW

Our results extend the existing literature by documenting food insecurity disparities in diverse subgroups of SMW, using a comprehensive, USDA endorsed measure of food insecurity. Compared to exclusively heterosexual women, lesbian WSW, bisexual WSW, and heterosexual WSW were 34–52% more likely to report experiencing past 12-month food insecurity. Alarmingly, SMW were 50–84% more likely to experience at least one period during the past 12-months where eating patterns were disrupted and food intake was reduced due to lack of money or other resources (i.e., severe food insecurity). This study is among the first to document food insecurity disparities in heterosexual WSW—an understudied subgroup of SMW.

Our results differ from others where significant differences in food security were not indicated between sexual minority and heterosexual adults in bivariate analyses (12% vs. 11%, *p* = n.s.), nor multivariable models (aOR = 1.19, *p* = n.s.), nor by gender [10]. This may reflect a measurement issue; respondents in these studies were asked to report only on past 30-day experiences of food insecurity [10]. In the general US population, approximately 5.9% of households report past 30-day food insecurity; however, over 11.1% report experiencing food insecurity over the past 12 months [1]. On average, food insecure households experience food insecurity for 7 months out of the year [1]; as such, surveys using 30-day recall periods may underestimate food insecurity disparities. By using a comprehensive measure of food security with a 12-month recall period, our study depicts the extent of food insecurity and disparities experienced annually by SMW.

### Food assistance use in SMW

Existing studies indicate that SMW are 30–70% more likely to receive SNAP benefits than heterosexual adults [9, 10]; however, our study did not evidence differences in SNAP use by sexual orientation. In contrast, lesbian WSW were 89% more likely and heterosexual WSW were 43% more likely to report past 12-month use of emergency food assistance (e.g., food pantries and soup kitchens) than exclusively heterosexual women. Emergency food participation has not been explored in previous population-based studies of food insecurity in sexual minority populations; consequently, this finding represents a new addition to the food insecurity and sexual minority health disparities literatures.

It is concerning that SMW are more likely to use emergency food assistance, but not more likely to use SNAP, despite evidencing disparities in food insecurity. SNAP participation reduces food insecurity [36, 37]; as such, increasing SMW’s SNAP participation may alleviate disparities. One explanation for SMW’s underutilization of SNAP is that SMW women may earn too much to qualify for SNAP, but not enough to afford food. SNAP guidelines require that a recipient’s gross income fall below 130% FPL (approximately $15,800 annually). However, a meta-analysis of earnings and wages suggests that, on average, lesbians earn 9% more than heterosexual women [38]. For low income SMW, this “lesbian premium” (i.e., a 9% wage differential) could be great enough to exclude SMW from qualifying for SNAP while leaving a reduced amount of income to afford food without federal assistance. “Working poor” Americans are more likely to recurrently use community-based emergency food assistance [39], which may explain SMW’s prevalent emergency food assistance use.

### Public health implications

Warnecke’s model of population health suggests that multilevel interventions are needed to reduce health disparities experienced by minority populations [8]. These may include community-based interventions; local, state, or federal policy; or efforts to produce social norms change [8]. In light of this model and study results, several multilevel community-based and policy solutions may be implemented to reduce food insecurity in SMW. At the local level, increasing access to local food assistance is necessary to support food insecure SMW who do not qualify for SNAP benefits. However, food pantries may not an accessible food source for all SMW. Regional studies suggest that many community-based emergency food assistance programs are religiously affiliated [17, 40], which may present a barrier for SMW who feel uncomfortable accessing religiously-affiliated food pantries due to fear of religiously-based discrimination [41]. To our knowledge, no studies have explicitly investigated the experiences of SMW who access food pantries. However, in a recent qualitative study of food insecure transgender and gender non-conforming (TGNC) individuals, respondents were less likely to seek food assistance in their local communities due to fear of gender- and sexual orientation-based stigma and discrimination from religiously-affiliated food pantries [42]. One solution is the rise of LGBT-specific food pantries sponsored by community-based organizations in major metropolitan areas. However, it is unclear how many food insecure SMW know about or access these pantries, nor how accessible they are for SMW living in rural and suburban areas. Mixed-methods studies investigating local factors that exacerbate and alleviate food insecurity for SMW (e.g., food pantries, community networks, and individual-level coping strategies) may inform the improvement of existing food pantries or development of newer methods. These may include locally organized food sharing communities via online social platforms that proactively engage vulnerable, food insecure SMW.

It is not enough, however, to increase access to emergency food resources. Decreasing food insecurity in SMW also requires increasing SMW’s participating in food insecurity-alleviating programs. Increasing SNAP participation in food insecure SMW may be challenging, as limits on SNAP benefits may disproportionately disadvantage SMW. In 2018, the USDA proposed a rule that would limit access to SNAP benefits to able-bodied adults without dependents (ABAWD) having trouble securing employment [43]. This is problematic for many SMW who are not protected from sexual orientation-based employment discrimination. One in 10 LGBT workers have left a job due to employment discrimination and almost 1 in 7 fear termination due to their sexual orientation [44]. Evidence from the general population suggests that expansion of work requirements eliminates SNAP benefits for ABAWD by nearly one-third [45]. In light of workplace and hiring discrimination, the proposed changes to SNAP could disproportionately affect SMW. Without SNAP to supplement food supplies, it is possible that more SMW will experience food insecurity and negative sequelae.

Decreasing food insecurity in SMW also requires addressing determinants of economic instability. Employment discrimination results in destabilized employment histories and lowered wages for SMW, which increases risk for poverty and food insecurity. Preventing employment discrimination for SMW requires instituting federal and/or state nondiscrimination laws that protect sexual minorities. To date, most employment nondiscrimination policies are state-based, creating a patchwork of protections for SMW. More recently, a coalition of 180 businesses guided by the Human Rights Campaign pledged support for the federal Equality Act; legislation that would prohibit discrimination based on sex, sexual orientation, and gender identity across public accommodations, employment, housing, education, and federal funding [46]. While promising, the Equality Act has yet to pass both the House *and* Senate [46]. Decreasing food insecurity disparities and increasing health equity for SMW, requires public health researchers and practitioners to lead policy efforts that promote sexual minority-supportive workplaces.

### Limitations

NHANES assesses gender with a single interviewer-administered item as part of household screening. Interviewers are asked to make an assessment of a respondent’s gender as male or female based on physical characteristics, and to ask respondents if they are not clear when assigning a respondent’s assumed gender. This is not congruent with best practice [47] and is problematic. Interviewers may erroneously assign a respondent’s gender, leading to under or overestimates of women in the sample. NHANES also does not assess transgender or gender nonconforming (TGNC) identity [48], which may lead to TGNC respondents being misclassified as cisgender male or female. This is problematic as TGNC populations may differently experience social and economic resource loss due to heterosexism, homophobia, cissexism, and transphobia, increasing their risk for food insecurity. NHANES’ sexual identity measures are double-barreled; each identity response (e.g., “lesbian”) is paired with a statement about sexual attraction (e.g., “sexually attracted to females”). This may conflate responses as individuals must choose a single response that comprises multiple aspects of their sexual orientation in a single question. Also, a considerable number of respondents did not complete the NHANES’ sexual behavior questionnaire, which may influence food insecurity estimates in sexual minority populations. Individuals who responded to sexual identity questions as “something else”, “other”, “don’t know”, or “refused” were excluded in this study as best practices for studying sexual minority health disparities caution against including respondents who refuse to answer sexual orientation questions due to potential confounding [24]. Finally, NHANES asks sexual orientation questions only for women up to age 59; estimates of food insecurity may differ in older SMW.

## Conclusion

This study provides the first population-level evidence of food insecurity disparities in SMW using a comprehensive measure of past 12-month food security. Compared to exclusively heterosexual women, SMW are more likely to experience disruptions in quality, desirability, type of food, and reduced food intake. SMW’s increased rate of food insecurity may contribute to chronic disease disparities, including cancer [49] and diabetes [50], evidenced in this population. SMW in our study were not more likely than exclusively heterosexual women to use SNAP; however, they were more likely to report past 12-month use of emergency food assistance, including food pantries. While studies find that SNAP reduces recipients’ food insecurity, emergency food assistance does not. As such, increasing SMW’s using of food insecurity-alleviating programs—including SNAP—may be necessary to decrease disparities in this population.

## Data Availability

The datasets analyzed during the current study are available from the Center for Disease Control and Preventions’ National Center for Health Statistics. https://www.cdc.gov/nchs/nhanes/index.htm

## Abbreviations

ABAWD: Able-bodied adults without dependents
CI: Confidence Interval
GED: General Education Diploma
NHANES: National Health and Nutrition Examination Survey
*P*: *P = value*
FPL: Federal poverty level
LR X2: Likelihood Ratio chi-squared test for proportions
PR: Prevalence Ratio
SNAP: Supplemental Nutrition Assistance Program
SMW: Sexual minority women
USDA: United States Department of Agriculture
US: United States
WSW: Women reporting lifetime sexual behavior with women
